# Assessment of Purity, Functionality, Stability, and Lipid Composition of Cyclofos-nAChR-Detergent Complexes from *Torpedo californica* Using Lipid Matrix and Macroscopic Electrophysiology

**DOI:** 10.1007/s00232-023-00285-x

**Published:** 2023-05-04

**Authors:** Orestes Quesada, Joel E. González-Nieves, José Colón, Rafael Maldonado-Hernández, Carol González-Freire, Jesús Acevedo-Cintrón, Irvin D. Rosado-Millán, José A. Lasalde-Dominicci

**Affiliations:** 1 Department of Physical Sciences, University of Puerto Rico, Río Piedras Campus, San Juan, PR USA; 2 Department of Chemistry, University of Puerto Rico, Río Piedras Campus, San Juan, PR USA; 3 Department of Biology, University of Puerto Rico, Río Piedras Campus, San Juan, PR USA; 4 Department of Pharmaceutical Sciences, Medical Sciences Campus, University of Puerto Rico, San Juan, PR USA; 5 Department of Biology, University of Puerto Rico, Ponce Campus, Ponce, PR USA; 6 Molecular Science Center, University of Puerto Rico, San Juan, PR USA; 7 Institute of Neurobiology, University of Puerto Rico, Medical Science Campus, San Juan, PR USA

**Keywords:** *Torpedo californica* nAChR, Two electrode voltage clamp (TEVC), Fluorescence recovery after photobleaching (FRAP), Ultra-Performance liquid chromatography mass spectrometry (UPLC-MS)

## Abstract

The main objective of the present study was to find detergents that can maintain the functionality and stability of the *Torpedo californica* nicotinic acetylcholine receptor (*Tc*-nAChR). We examined the functionality, stability, and purity analysis of affinity-purified *Tc*-nAChR solubilized in detergents from the Cyclofos (CF) family [cyclofoscholine 4 (CF-4), cyclofoscholine 6 (CF-6), and cyclofloscholine 7 (CF-7)]. The functionality of the CF-*Tc*-nAChR-detergent complex (DC) was evaluated using the Two Electrode Voltage Clamp (TEVC) method. To assess stability, we used the florescence recovery after photobleaching (FRAP) in Lipidic Cubic Phase (LCP) methodology. We also performed a lipidomic analysis using Ultra-Performance Liquid Chromatography (UPLC) coupled to electrospray ionization mass spectrometry (ESI–MS/MS) to evaluate the lipid composition of the CF-*Tc*-nAChR-DCs. The CF-4-*Tc*-nAChR-DC displayed a robust macroscopic current (− 200 ± 60 nA); however, the CF-6-*Tc*-nAChR-DC and CF-7-*Tc*-nAChR-DC displayed significant reductions in the macroscopic currents. The CF-6*-Tc*-nAChR and CF-4-*Tc*-nAChR displayed higher fractional florescence recovery. Addition of cholesterol produced a mild enhancement of the mobile fraction on the CF-6-*Tc*-nAChR. The lipidomic analysis revealed that the CF-7-*Tc*-nAChR-DC displayed substantial delipidation, consistent with the lack of stability and functional response of this complex. Although the CF-6-nAChR-DC complex retained the largest amount of lipids, it showed a loss of six lipid species [SM(d16:1/18:0); PC(18:2/14:1); PC(14:0/18:1); PC(16:0/18:1); PC(20:5/20:4), and PC(20:4/20:5)] that are present in the CF-4-nAChR-DC. Overall, the CF-4-nAChR displayed robust functionality, significant stability, and the best purity among the three CF detergents; therefore, CF-4 is a suitable candidate to prepare *Tc*-nAChR crystals for structural studies.

**Graphical abstract:**

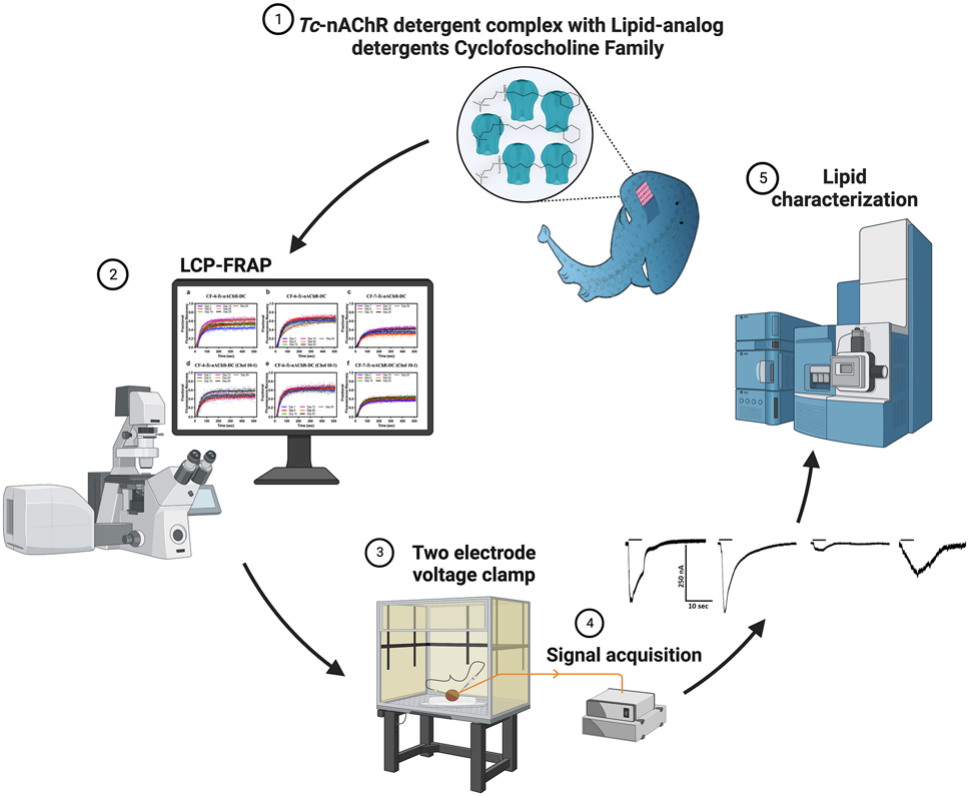

## Introduction

The nicotinic acetylcholine receptors (nAChRs) are integral membrane proteins and are one of the most characterized ligand-gated ion-channels super family. The nAChRs are pentameric proteins with a different assembly from a pool of seventeen homologous polypeptides (subunits): α1-α10, β1-β4, γ, δ, and ε, (Gotti and Clementi 2004; Zoli et al. 2015). The nAChRs are widely distributed in different tissues in mammals and other animal species and have been implicated in various neurological diseases. These include but are not limited to congenital myasthenic syndromes, tobacco addiction, Alzheimer’s disease, Parkinson’s disease, schizophrenia, epilepsy, Turret’s syndrome, inflammation, and more recently COVID-19 infection, (Gotti and Clementi 2004; Zoli et al. 2015; Lucatch et al. 2018; Farsalinos et al. 2020; Mashimo et al. 2020; Bekdash 2021; Hollenhorst and Krasteva-Christ 2021; Recio-Barbero et al. 2021; Jankauskaite et al. 2022; Tiepolt et al. 2022).

There is an overwhelming need of high-resolution structures for each nAChR subtype and their binding sites, to facilitate the design of new selective therapeutic drugs to various regions of the extracellular domain and other domains of these receptors (e.g., binding sites, pore, or putative allosteric sites). The first and only X-ray structure of the heteromeric neuronal α4β2-nAChR was reported in 2016 (Morales-Perez et al. 2016). The structural data from this study were collected from one crystal out of thousands of crystals screened (personal communication with Dr. Morales-Pérez). The difficulties of reproducing high-quality α4β2-nAChR crystals led to the use of Cryo-EM, and in 2018, two different stoichiometries of the same α4β2-nAChR were determined (Walsh et al. 2018). Although these α4β2-nAChR structures have provided substantial information about nicotine binding, cholesterol-binding, subunit stoichiometry, and overall oligomerization, these are low-resolution structures (~ 3.9 Å). The principal limitations of the α4β2-nAChR structures (X-ray and CryoEM) structures are (1) their inability to reproduce high-quality crystals for drug discovery studies and (2) the very limited information for structure-based drug design that they provide. Recently, three additional Cryo-EM structures have emerged: (1) the α3β4 in nanodiscs at 4.58 Å resolution (Gharpure et al. 2019), (2) the *Torpedo californica* (*Tc*) (muscle-type) nAChR in the closed state (Rahman et al. 2020), and (3) the α7-nAChR in three different channel conformation states resting-like closed-channel state, with the positive allosteric modulator PNU-120596 and agonist epibatidine/α7-nAChR complex with average resolution of 3.6 Å (Noviello et al. 2021). Some of the limitations of the α7-nAChR structure are that the open conformation of the channel was a proxy and that the position of the PNU-120596 in the proposed binding site was not defined due to limited resolution of the CryoEM images.

The first obstacle in achieving high-resolution, X-ray structures of the nAChRs is the preparation of milligram amounts of pure, homogeneous, functional, and stable nAChR-detergent complexes (nAChR-DCs). Other difficulties in crystalizing nAChRs are: (1) heterogenic pentamers, (2) multiple stoichiometries, (3) pseudosimetry of heteropentamers, (4) glycosylation of extracellular domains with diverse sugar compositions, (5) large intracellular domains (M3-M4 loop) with disordered structure, and (6) different conformations (Asmar-Rovira et al. 2008; Delgado-Vélez et al. 2021). Along these lines, the preparation and the reproducibility of nAChRs protein crystals suitable for X-ray diffraction studies have become remarkably challenging experimentally and the foremost obstacle to attaining high-resolution structures.

In the present study we prepared *Tc*-nAChR-DCs using three lipid-analog detergents bearing a six-member carbon ring at the tail end of a homologous series of phosphocholine (Table 1). We evaluated purity, stability, functionality, and lipid composition of the nAChR-DCs. We adopted the lipidic cubic phase (LCP) as a lipid matrix suitable for the stable relocation of the solubilized and affinity-purified *Tc*-nAChR-DC to assess stability (Padilla-Morales et al. 2015, 2016). LCP has become an efficient matrix for harvesting protein crystals of different molecular weights, achieving the deposit of over 120 unique protein structures according to the Protein Data Bank (Landau and Rosenbusch 1996). To assess protein-detergent complex stability we used LCP-Fluorescence Recovery After Photobleaching (LCP-FRAP) assay during a 30-day period to measure parameters, such as the mobile fraction and diffusion coefficient which correlate with protein stability and aggregation (Padilla-Morales et al. 2015, 2016). We examined the *Tc*-nAChR-DC ion-channel functionality using macroscopic ion-channel behavior in *Xenopus laevis* oocytes via the Two Electrode Voltage Clamp (TECV) technique. In addition, the nAChR-DC lipid composition was accessed via Ultra-Performance Liquid Chromatography (UPLC) couple to Quadrupole Time-of-Flight (QTOF) mass spectrometry. The objective of this study was to investigate and characterize the effect of detergents in *Tc*-nAChR. Furthermore, we studied the effect of cholesterol in regulating the *Tc*-nAChR-DC complex stability. Assessing lipid-analog *Tc*-nAChR-DC functionality and stability and characterizing the conditions where the receptor resembles and behaves as its native environment might be fundamental to future structural studies of the nAChR and other membrane proteins.

**Table 1:** Lipid-analog detergents Cyclofoscholine Family

Detergent	CMC	Solubilization	Structure
Cyclofoscholine-4	8.45 mM	> 20%	
Cyclofoscholine-6	2.68 mM	> 20%	
Cyclofoscholine-7	0.62 mM	> 20%	

## Materials and Methods

### Materials

All reagents were purchased from Sigma-Aldrich unless otherwise specified. The lipid-like cyclic detergent Cyclohexyl-1-Butylphosphocholine family 4-Cyclohexyl-1-Butylphosphocholine [Cyclofoscholine-4 (CF-4)], 6-Cyclohexyl-1-Hexylphosphocholine, [Cyclofoscholine-6 (CF-6)] and 7-Cyclohexyl-1-Heptylphosphocholine [Cyclofoscholine-7 (CF-7)] at purity 98%, were obtained from Anatrace (Maumee, OH, USA), Table 1.

### Preparation of Crude Membrane

The nAChR was extracted from rich membranes from the electric organ of (*Tc*) (Aquatic Research Consultants, San Pedro, CA), according to the procedure of Asmar-Rovira (Asmar-Rovira et al. 2008) and with minor modification as described previously by Padilla (Padilla-Morales et al. 2015, 2016) and Quesada (Quesada et al. 2016). To avoid possible seasonal changes in lipid content, all the experiments were performed with the same *Tc* electric organ.

We incubated 200 g of *Tc* tissue with 200 ml of buffer H (100 mM NaCl, 10 mM Sodium Phosphate, 5 mM EDTA, 5 mM EGTA, 5 mM DTPA, 0.02% Sodium Azide, pH 7.4) mixed with 200 μl of phenyl methane sulfonyl fluoride (PMSF) and 0.187 g of Iodoacetamide, in a cool room.

### Affinity-Column Purification of Solubilized Tc-nAChR

The solubilized nAChR was purified by means of affinity-column using the protocol of Padilla and Quesada (Cheng et al. 1998; Cherezov et al. 2008; Padilla-Morales et al. 2015; Padilla-Morales et al. 2016; Quesada et al. 2016). Briefly, the crude membranes were thawed and mixed with a 10% (w/v) detergent solution and DB-1X Buffer (100 mM NaCl, 10 mM MOPS, 0.1 mM EDTA, 0.02% NaN3) for a final concentration of detergent 1–2%. The DB-1X buffer was added first, followed by the detergent, and finally the crude membranes, which were added drop by drop. This solution was shaken slowly for 1 h. and then centrifuged for 1 h. at 40,000 rpm and 4 °C. The supernatant was extracted and used immediately for the affinity-column purification. Approximately 12 mL of previously prepared bromoacetylcholine affinity resin (Bio-Rad Laboratories, Hercules, CA) in a 1.5 × 15 cm Econocolumn (Bio-Rad Laboratories, Hercules, CA) was drained of storage buffer (40% Sucrose, 2 mM PMSF) and was conditioned with 50 mL of ultrapure water and 50 mL of 1.5 critical micelle concentration (CMC) detergent buffer before the supernatant prepared previously was added to the column. The column was washed with 50 mL of 1.5 CMC detergent buffer before the nAChR was eluted with 50 mL of elution buffer. The sample was then concentrated using centrifuge filter with a 100 K cutoff (Amicon Ultra Centrifugal Filters Ultracel 100 K, Millipore Co., Billerica, MA) and run through a P-10 desalting column (GE Healthcare, Uppsala, Sweden) to remove the carbamylcholine ligand. The sample was eluted with 5 mL of 1.5 CMC detergent buffer and finally concentrated to 250 μL. Protein concentration was determined using a BCA Protein Concentration Assay (Pierce Biotechnology, Rockford, IL) followed by sodium dodecyl sulfate polyacrylamide gel electrophoresis (SDS-PAGE), which was run to verify receptor purity.

### Sodium Dodecyl-sulfate Polyacrylamide Gel Electrophoresis (SDS-PAGE)

Samples were prepared by mixing 20 μl (1 μg/uL) of purified protein with 20 μL of Lamlli 2 × Buffer. Gel electrophoresis was performed by loading 20 μL of protein in Criterion TGX precast gels. The samples were run in duplicates for 2 h at 120 Volts. Gel was stained with 1X Coomassie Blue and left overnight. After 10–12 h, gel was washed with destaining solution (10% acetic acid, 40% water, and 50% methanol), for 3 h., followed by three washes with distilled water.

### LCP-nAChR-Detergent Complex Mobility Assay Using FRAP

FRAP experiments were performed according to the conditions and protocols described by Cherezov (Cherezov et al. 2008) with the minor modifications presented by Padilla-Morales (Padilla-Morales et al. 2016). Briefly, the affinity-purified nAChR-detergent complex was incubated with alpha Bungarotoxin (αBTX) conjugated with Alexa 488 in a 1:2.5 ratio, respectively, for 2 h. in the dark at 4.0 °C. The nAChR-detergent complex- αBTX was mixed with molten monoolein (1-oleoyl-*rac*-glycerol in a 2:3 volume ratio), using a lipid mixer (Hamilton Syringe) and mixed until clear (Cheng et al. 1998). The resulting mixture was placed on a 75 mm × 25 mm slide coated with pre-punched holes of 7 mm diameter and 50 μm thickness (3 M 9482PC), and the formed wells were then covered by pressing a coverslip against the slide and flattened with a rubber roll (Cherezov et al. 2002; Caffrey and Cherezov 2009). The experimental procedure was conducted in a controlled environment maintaining the humidity between 40 and 50% at any time.

### Lipidic Cubic Phase-Cholesterol Mixture

For any of the assays in which cholesterol was used to supplement monoolein, we used the commercially available Monoolein (and Cholesterol (H200) mixture (Anatrace), and the ratio was 1-oleoyl-rac-glycerol (10 parts): Cholesterol (1 part). The rest of the procedure for cholesterol assays was one as described above, (Tyler et al. 2015).

### FRAP Instrument Setup and Data Collection

Data collection was performed as described in Cherezov et al. (2008) and Padilla-Morales et al. (2016). Briefly, all data were collected using a Zeiss LSM 510 confocal microscope. Fluorescence baseline was established by pre-bleach images in several areas: 75% of laser bleaching power followed by a sequence of 500 images scanning at 2.6% power with a 600 ms laser scanning delay. All images obtained were processed using the LSM 510 Meta ZEN software. Each sample slide was monitored for 30 days at intervals of 5 days. The data were integrated within a 14.0 µm diameter circular region of interest (ROI_1_) and corrected and normalized by another 14.0 µm circular region of interest (ROI_2_) positioned near the bleached ROI_1_.

Fluorescence intensity was adjusted by dividing the integrated intensity value of ROI_1_ in the bleached spot by the average integrated intensity of ROI_2_. As described by Cherezov’s research group (Cherezov et al. 2008), the fractional fluorescence recovery curves F(t) were calculated using the following equation.

1 $$F\left(t\right)=\left[({f}_{t}-{f}_{0})/{f}_{\infty }-{f}_{0}\right],$$

where F_(*t*)_ is the corrected fluorescence intensity of the bleached spot, f_0_ is the corrected/normalized fluorescence intensity of the bleached spot during the 600 ms after bleaching, and ${f}_{\infty }$ is the average of corrected fluorescence intensity in the five pre-bleached images. Fractional mobility values were obtained by calculating the average of the last 50 values of F(t). The fractional fluorescence recovery curves were fitted with a one-dimensional exponential Plot (Eq. 2).

2 $$f\left(t\right)=\sum_{i=1}^{n}{A}_{i }\left(1-{exp}^{\left(-Kt\right)}\right)+B,$$

where *A*_*i*_ is the amplitude of each component, *K* is a constant related to the degree of bleaching, *t* is time, and *B* is a constant related to the mobile fraction of receptors (Axelrod et al. 1976).

The fractional fluorescence recovery curves were fitted with a one-dimensional equation (one Phase Exponential Plot) provided by Graph Pad statistical analysis software. The Diffusion coefficient value was calculated using Eq. 3; where R is the half width at half maximum of the Gaussian function [R = r(2ln2)^0.5^] and K is a constant calculated using Eq. 2 as described by Cherezov (Pucadyil and Chattopadhyay 2006).

3 $$D=\left[{R}^{2}/4K\right]$$

### Injection With Crude Membrane or nAChR-Detergent Complex into Oocytes and Two Electrode Voltage Clamp Assays

We followed the original protocols by Andrés Morales et al. 1995; Ivorra et al. 2002; Andrés Morales et al. 2006) with the modifications described in Padilla and Quesada (Padilla-Morales et al. 2016; Quesada et al. 2016), Briefly, the *Xenopus leavis* oocytes used were in developmental stage V or VI. Each oocyte was injected with 50 nL of a preparation of 6 mg/mL of crude membrane or 3 mg/mL of 1.5-fold CMC nAChR-detergent complex, according to the CMC of the detergent used in the purification of the *Tc* nAChR. Subsequently, oocytes were incubated at 18 °C for 16–36 h in ND-96 solution containing 96 mM NaCl, 2 mM KCl, 1.8 mM CaCl_2_, 1 mM MgCl_2_, 5 mM HEPES, 2.5 mM Na-pyruvate supplemented with gentamicin (50 mg/mL), tetracycline (50 mg/mL), and theophyline (0.5 mM); and adjusted to a pH of 7.6 with NaOH.

### Lipid Extraction and Separation by High Performance Thin Layer Chromatography

The lipid extraction and separation were conducted according to Quesada.^24^ Briefly, the purified *Tc* nAChR-DCs were lyophilized overnight and subjected to lipid extractions using B&D methods in the presence of butylated hydroxytoluene (BHT; 2.9 × 10 − 5 M), followed by 3.5 h of reflux with MeOH/HCl or MeOH/1N KOH for complete phospholipid hydrolysis. Phospholipids species were resolute using commercially available high-performance thin layer chromatography (HPTLC) plates (20 × 20 cm) from Whatman, Fisher Scientific, MA, USA. The plate containing the samples was developed in chloroform: methanol: ammonium hydroxide (60:35:5).

### Solid Phase Extraction of Lipid Samples (Pre-Cleaning)

The separation of lipids previous to mass spectrometry analysis was accomplished using an aminopropyl extraction column (particle size 40 μm; Agilent Bond Elut NH_2_, Agilent Corporation, Palo Alto, CA, USA), as described by Quesada (Quesada et al. 2016). Briefly, the dry *Tc* nAChR-detergent complex was dissolved in CHCl_3_ and uploaded to Bond Elut NH_2_, following the manufacturer’s indications. The column was conditioned by passing 6 mL of hexane, then 200 μL CHCl3 lipid extract was loaded followed by a sequential elution with four different eluents: 2 mL of CHCl_3_, 3 mL of diethyl ether with 2% acetic acid, 3 mL MeOH, and a final 3 mL of 0.05 M ammonium acetate in chloroform/methanol plus 2% (v/v) 28% aqueous ammonium solution. The four fractions collected contained the non-polar lipids and cholesterol, non-esterified fatty acids, non-acids phospholipids, and acidic phospholipids, respectively.

### Tc Membrane and nAChR Detergents Cholesterol Quantitation

The cholesterol extraction, isolation, and quantification were achieved according to Quesada (Quesada et al. 2016). Briefly, the cholesterol extracted from *Tc* membrane and nAChR-DC were isolated from the rhodamine 6G stained silica gel G plates and further quantified using the Wako cholesterol E-Kit (Wako Chemicals, Richmond, VA, USA).

### Analysis of Phospholipid Molecular Species by Ultra-Performance Liquid Chromatography (UPLC) Coupled to Electrospray Ionization Mass Spectrometry (ESI–MS/MS)

Phospholipids isolated from the Bond Elut NH2 cartridge, as mentioned above, were analyzed using UPLC ESI–MS/MS or MSe with an ACQUITY UPLC coupled to an XEVO G2S quadrupole-time-of-flight mass spectrometry (QToF) from Waters Corp. using BEH HILIC (1.7 μm, 2.1 mm × 100 mm) column as described by Quesada, (Quesada et al. 2016). Briefly, the sample was run using the following conditions for UPLC and QToF; the mobile phase A was 10 mM ammonium acetate in water at pH 3, adjusted using formic acid, and mobile phase B was acetonitrile. The gradient was as follows: 0–0.1 min, 100% B; 0.1–0.5 min, 92% B; 0.5–15 min, 80% B; and then back to 100% B at 15.1 min. to re-equilibrate the column for about 1 min. The injection volume was 0.5 μL, and the flow rate was 0.3 μL/min. ESI analysis was performed in positive resolution mode using the MSe continuum method. The instrument was calibrated with a sodium iodide standard solution (2 μg/μL) in 2 propanol/water (50:50). The voltages used were: capillary 3 kV, sampling cone 75 kV, and source offset 40 kV. The source temperature was 100 °C, and the desolvation temperature was 350 °C. The gasses’ flows were: cone 50 L/h and desolvation 800 L/h. The acquisition time was 15 min, mass range 50 to 1,100 Dalton, and the collision energy ramp in range of 20 V to 30 V. Leucine enkephalin (2 ng/μL) was used as a reference; a capillary voltage of 2 kV and a flow rate of 3.0 μL/min were employed.

### Statistical Analysis

All data were processed and statistical analyses were conducted using the GraphPad Prism 9 software (GraphPad Software, San Diego, CA, www.graphpad.com). All samples were analyzed separately using one-way ANOVA followed by Tukey’s multiple comparison test. The activation and deactivation kinetics of CF-*Tc*-nAChR-DCs were analyzed statistically using a t-test Mann Whitney comparing all the different CF-*Tc*-nAChR-DCs to crude membranes.

## Results

### Effect of Cyclofoscholine Detergent on the Stability of the Tc-nAChR-Detergent Complex in LCP-FRAP

We used FRAP to assess the stability of the *Tc*-nAChR-DCs in LCP matrix. The use of LCP was introduced by Landau and Rosenbusch and by Rummel (Landau and Rosenbusch 1996; Rummel et al. 1998). This approach allows measuring the mobile fraction of the *Tc*-nAChR-DC in LCP by FRAP. Our group previously determined the fraction fluorescence recovery and mobile fraction of several lysophospholipids and cholesterol-analogs detergents for the *Tc* nAChR-Alexa 488-DC (Padilla-Morales et al. 2015, 2016). We also evaluated the effect of the addition of cholesterol in the LCP matrix in the stability of CF-nAChR-DCs. In order to evaluate the stability of the CF-nAChR-DCs the fractional fluorescence recovery, mobile fraction, and diffusion coefficient were determined for a period of 30 days at intervals of 5 days.

**Fig. 1:**
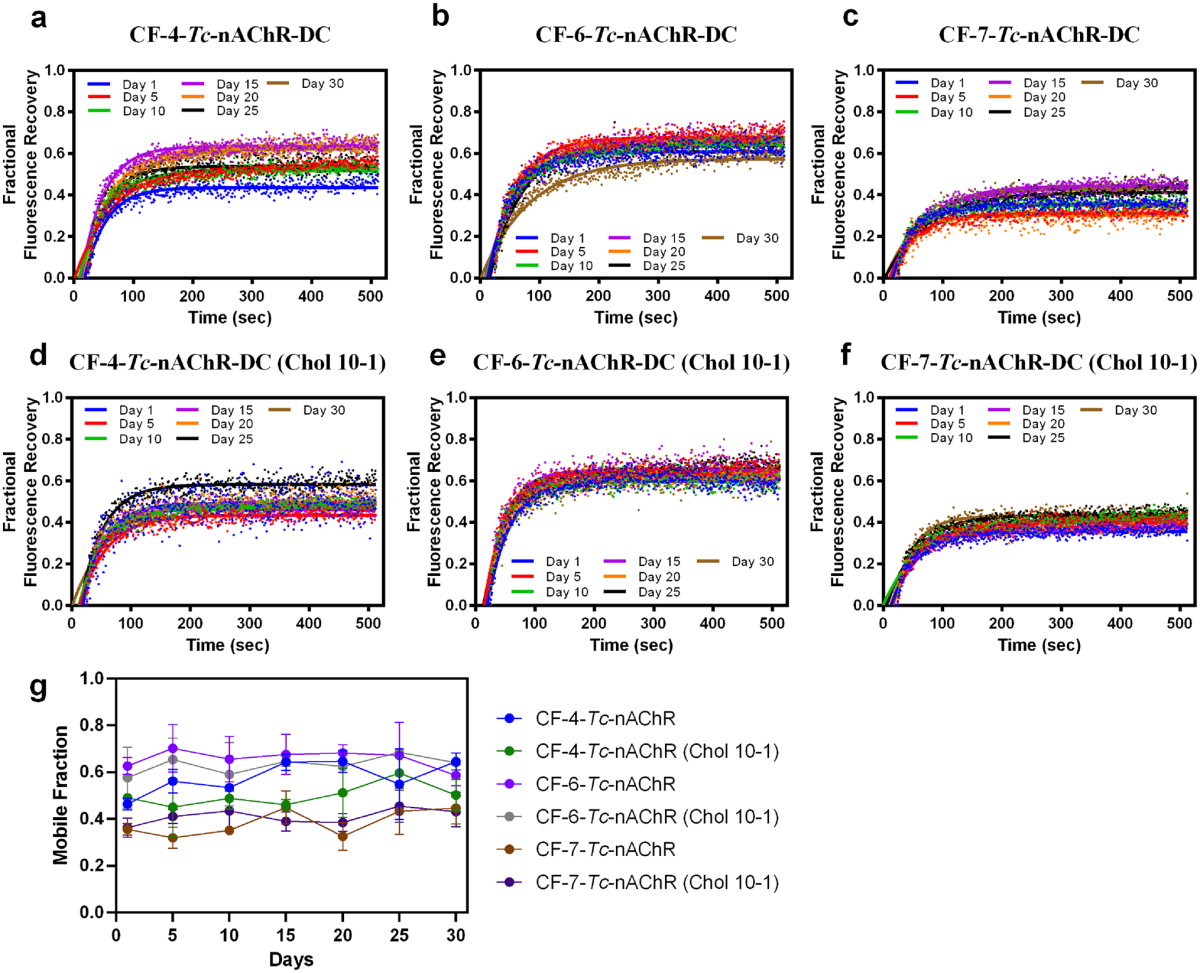
Fractional Fluorescence Recovery CF-*Tc*-nAChR-DC Family for 30 days. FRAP experiments were recorded for each of the CF detergents and fractional fluorescence recovery was recorded every five days for thirty days. Experiments were performed in triplicates (*n* = 3), with the average of three recoveries from different RO1s. (**a**) CF-4-*Tc*-nAChR-DC, (**b**) CF-6-*Tc*-nAChR-DC, (**c**) CF-7-*Tc*-nAChR-DC. The panels (**d**), (**e**), and (**f**) represent the same CF-*Tc*-nAChR-DC loaded to LPC doped with cholesterol in 10:1 ratio, respectively. The mobile fraction for were obtained by averaging the last twenty points of the fractional recovery obtained for each of the conditions above (**g**)

Figure 1 shows the fractional fluorescence recovery for CF-4, CF-6, and CF-7, panels (a), (b), and (c), respectively. Each CF-*Tc*-nAChR-DC presents differences in the value of the fractional fluorescence recovery measured at intervals of 5 days. The variability during the 30 days is different for the three detergents evaluated, measured in the plateau at 500 s, with CF-6 being the one with the least variability (0.60–0.72), followed by CF-7 (0.30–0.50), and CF-4 having the greatest (0.45–0.70). However, CF-4 and CF-6 presented different fluorescence recovery values of 0.64 and 0.55, respectively, at day 30 whereas CF-7 presented a very lower mean value of 0.47 at day 30.

Since nAChRs have been shown to have cholesterol-modulated activity and stability, we used a mixture of monoolein and cholesterol in a 10:1 ratio to perform FRAP assays. Figure 1 middle panels (c), (d), and (e) present the fractional fluorescence recovery as a function of time for the three CF-*Tc*-nAChR-DC cholesterol supplemented LCP. At first glance, a decrease in the variability of the average values of fractional fluorescence recovery measured at the plateau with intervals of 5 days is observed for the three detergents studied. However, the average fractional fluorescence recovery value at 30 days was slightly lower for CF-4-*Tc*-nAChR-DC and CF-6-*Tc*-nAChR-DC when compared to the values obtained for pure monoolein. The exception was CF-7-*Tc*-nAChR-DC, which maintained a similar value on practically all the days tested. All CF-*Tc*-nAChR-DCs tested presented diffusion coefficient values in ranges previously observed for this type of protein in LCP (Cherezov et al. 2002; Padilla-Morales et al. 2016). The diffusion coefficient values determined for CF-4-Tc-nAChR-DC, CF-6-Tc-nAChR-DC, and CF-7-Tc-nAChR-DC were 1.33 × 10 − 8 cm2/sec, 1.13 × 10 − 8 cm2/sec, and 1.25 × 10 − 8 cm2/sec, respectively. Although these three detergents showed very similar diffusion coefficients, when the FRAP assay was carried out in LCP supplemented with cholesterol in a ratio of 10:1 (monoolein:cholesterol), the CF-4-Tc-nAChR-DC and CF-6-Tc-nAChR-DC displayed significant increases in their diffusion coefficient compared to their LCP non-cholesterol counterpart, 1.48 × 10 − 8 cm2/sec, and 1.70 × 10 − 8 cm^2^/sec, respectively. The CF-7-Tc-nAChR-DC exhibited a modest increase in the diffusion coefficient (1.29 × 10 − 8 cm^2^/sec).

Based on our mobile fraction analysis presented in Fig. 1 panel g, CF-7 with and without cholesterol exhibited a significant decrease over the 30-day period, with an average of 38% and 40% decrease in mobile fraction, respectively, compared to CF-6 with and without cholesterol, which had the highest average mobile fraction of 70% and 68%, respectively, and did not show significant changes between them. In contrast, CF-4 showed a linear increase in mobile fraction over the 30-day period from 46 to 65% without cholesterol and 45% to 50% for CF-4 with cholesterol. According to our results, the length of the carbon chain in each detergent significantly affected the mobile fraction in the LCP. We observed that detergents with longer carbon chains, such as CF-6 and CF-7, showed a lower mobile fraction compared to CF-4. This is because detergents with longer carbon chains have a higher affinity for molecules such as cholesterol, which plays a crucial role in reducing molecular mobility in the LCP. The interaction between detergents and cholesterol may lead to an increase in the rigidity of the cell membrane, resulting in a decrease in molecular mobility in the LCP. In addition, the presence of cholesterol in the membrane can lead to the formation of ordered and disordered lipid domains, which can also influence the mobility of molecules in the LCP. It is important to note that changes in the mobile fraction in the LCP can be an indicator of changes in the organization and composition of the membrane. Therefore, these results may have important implications for understanding cellular dynamics and molecular interactions in different types of cells and biological systems.

### Phospholipid Molecular Species of the CF-Tc-nAChR-DCs

Previous studies in our laboratory determined the endogenous lipid composition of the native *Tc* electric organ and of different complexes of *Tc*-nAChR with lipid-like detergents, and a correlation was made with their activity measured with two electron voltage clamp (TEVC) (Quesada et al. 2016). We used the same approach here in order to evaluate the composition of phospholipid molecular species in CF-*Tc*-nAChR-DCs and compare it with those detergents previously studied as a way to explain stability and functionality of the *Tc*-nAChR-detergent complex. Table 2 presents the different phospholipids molecular detected in the ESI positive mode for the three CF-*Tc*-nAChR-DCs previously mentioned. Only zwitterionic molecular species were detected under the experimental conditions used, such as sphingomyelin and alkenyl phosphatidylcholine. We did not detected measurable levels of any anionic molecular species even in the ESI negative mode. We have the same situation in a previous study using the phospholipid-analog detergents alkylphosphocholine (FC) and lysofoscholine (LFC) families of detergents (Quesada et al. 2016). For those two families of detergents, we also observed the same exclusion of negatively (acid-rich) phospholipids in the nAChR-DCs. There is no simple explanation for the lack of negatively charged lipids in the nAChR-detergent complexes, however, we hypothesize that these are not essential for the formation of stable mixed micelles containing appropriate levels of protein, lipid, and detergent during solubilization. In this regard, due to its smaller headgroup, PA might not allow for appropriate micellar curvature and those with negatively charged headgroups could also be overwhelmed by the larger fraction of cationic species in Tc tissue. Nevertheless, their absence would appear to indicate that these acidic phopholipids are not necessary for the presence of a nAChR protein in the respective DC that is capable of specifically binding to carbamylcholine in affinity chromatography and to α-bungarotoxin. Along these lines, previous studies (Sunshine and McNamee 1992; Fong and McNamee 1987; Poveda et al., 2002) had demonstrated that negatively (acid-rich) phospholipids can modulate nAChR ion-channel function. The lack of these (acid-rich) phospholipids in the nAChR-DCs could also contribute to the reduced functional responses recorded in oocytes. Cross-correlation of the lipid species detected for the three CF-*Tc*-nAChR-DCs (Table 3) shows similarities in some molecular species. The CF4-*Tc*-nAChR-DC has the highest number of retained lipid species, 13 in total, followed by the CF7-*Tc*-nAChR-DC and the CF6-*Tc*-nAChR-DC with 9 and 6 species, respectively. The three detergents manage to maintain only four identical lipid species: the Sphingomyelin SM (d18:1/24:1) in trace levels and the glycerophosphocholine PC (O16:1/18:0), PC (16:0/20:4), and PC (18:2/20:4), the latter having an abundance greater than 3%.

**Table 2:** Molecular species of phospholipid found in the extracted lipids from CF-*Tc*-nAChR-DCs using Bligh & Dyer extraction method. The samples from CF-*Tc*-nAChR-DCs were extracted using the Bligh & Dyer method and analyzed by UPLC ESI Q-Tof MS/MS in positive and negative resolution modes

m/z	Relative intensity	Specie	Exact mass	Formula		Error (ppm)	MS/MS product ions
4-Cyclohexyl-1-Butylphosphocholine (Cyclofos-4)							
675.5405	0.82	SM(d18:1/14:0)	675.5435		C_37_H_76_N_2_O_6_P	0.7	675,184
703.5699	79.58	SM(d16:1/18:0)	703.5748		C_39_H_80_N_2_O_6_P	7.0	703,184
703.5699	79.58	SM(d18:1/16:0)	703.5748		C_39_H_80_N_2_O_6_P	7.0	703,184
813.6845	100	SM(d18:1/24:1	813.6844		C_47_H_94_N_2_O_6_P	0.1	813,184
718.5788	12.46	PC(O16:0/16:1)	718.5745		C_40_H_81_NO_7_P	3.2	718,480,184
732.5510	11.18	PC(14:0/18:1)	732.5538		C_40_H_79_NO_8_P	3.8	732,468,184
746.6015	4.38	PC(O16:1/18:0)	746.6058		C_42_H_85_NO_7_P	5.8	746,184
746.6015	4.38	PC(O18:0/16:1)	746.6058		C_42_H_85_NO_7_P	5.8	746,184
760.5873	53.21	PC(16:0/18:1)	760.5851		C_42_H_83_NO_8_P	2.9	758,496,184
782.5666	7.64	PC(16:0/20:4)	782.5694		C_44_H_81_NO_8_P	3.6	782,496,184
806.5698	30.12	PC(18:2/20:4)	806.5694		C_46_H_81_NO_8_P	0.5	806,520,184
828.5534	6.45	PC(20:5/20:4)	828.5538		C_48_H_79_NO_8_P	0.5	828,184
828.5534	6.45	PC(20:4/ 20:5)	828.5538		C_48_H_79_NO_8_P	5.60.5	828,184
6-Cyclohexyl-1-Hexylphosphocholine (Cyclofos-6)							
813.6845	100	SM(d18:1/24:1	813.6844		C_47_H_94_N_2_O_6_P	0.1	813,184
718.5668	3.54	PC(O16:0/16:1)	718.5745		C_40_H_81_NO_7_P	3.2	718,480,184
746.6015	4.38	PC(O16:1/18:0)	746.6058		C_42_H_85_NO_7_P	5.8	746,184
746.6015	4.38	PC(O18:0/16:1)	746.6058		C_42_H_85_NO_7_P	5.8	746,184
746.6015	2.30	PC(16:0/18:1)	760.5851		C_42_H_83_NO_8_P	5.5	758,496,184
782.5666	6.83	PC(16:0/20:4)	782.5694		C_44_H_81_NO_8_P	3.6	782,496,184
806.5698	3.20	PC(18:2/20:4)	806.5694		C_46_H_81_NO_8_P	0.5	806,520,184
7-Cyclohexyl-1-Heptylphosphocholine (Cyclofos-7)							
675.5405	0.98	SM(d18:1/14:0)	675.5435		C_37_H_76_N_2_O_6_P	1.2	675,184
703.5699	79.58	SM(d16:1/18:0)	703.5748		C_39_H_80_N_2_O_6_P	7.0	703,184
703.5699	79.58	SM(d18:1/16:0)	703.5748		C_39_H_80_N_2_O_6_P	7.0	703,184
813.6845	100	SM(d18:1/24:1	813.6844		C_47_H_94_N_2_O_6_P	0.1	813,184
728.5246	28.35	PC(18:2/14:1)	728.5225		C_40_H_75_NO_8_P	2.9	728,504,184
746.6015	4.38	PC(O16:1/18:0)	746.6058		C_42_H_85_NO_7_P	5.8	746,184
746.6015	4.38	PC(O18:0/16:1)	746.6058		C_42_H_85_NO_7_P	5.8	746,184
760.5873	53.21	PC(16:0/18:1)	760.5851		C_42_H_83_NO_8_P	2.9	758,496,184
806.5698	3.20	PC(18:2/20:4)	806.5694		C_46_H_81_NO_8_P	0.5	806,520,184

**Table 3:** Cross-correlation of major lipid species present in the three CF-*Tc*-nAChR-DCs. The phospholipid composition of affinity-purified CF-*Tc*-nAChR-DCs was separated using HILIC column and analyzed by ESI–MS/MS

m/z (M + H)^+^	Specie	CF-4	CF-6	CF-7	R*
675.5405	SM(d18:1/14:0)			X	< 1%
703.5699	SM(d16:1/18:0)	X		X	> 3%
703.5699	SM(d18:1/16:0)	X		X	> 3%
813.6845	SM(d18:1/24:1	X	X	X	< 1%
718.5668	PC(O16:0/16:1)	X	X		< 1%
728.5246	PC(18:2/14:1)	X		X	< 1%
732.5510	PC(14:0/18:1)	X			> 3%
746.6015	PC(O16:1/18:0)	X	X	X	> 3%
746.6015	PC(O18:0/16:1)	X	X	X	> 3%
760.5873	PC(16:0/18:1)	X		X	~ 1%
782.5666	PC(16:0/20:4)	X	X		~ 1%
806.5698	PC(18:2/20:4)	X	X	X	> 3%
828.5534	PC(20:5/20:4)	X			> 3%
828.5534	PC(20:4/ 20:5)	X			~ 1%

R* Relative abundance in MS of crude membrane preparation. 3% major ones

### Effect of CF Family Detergent in the Phospholipids and Cholesterol to nAChR Ratio in the Detergent Complex

**Fig. 2:**
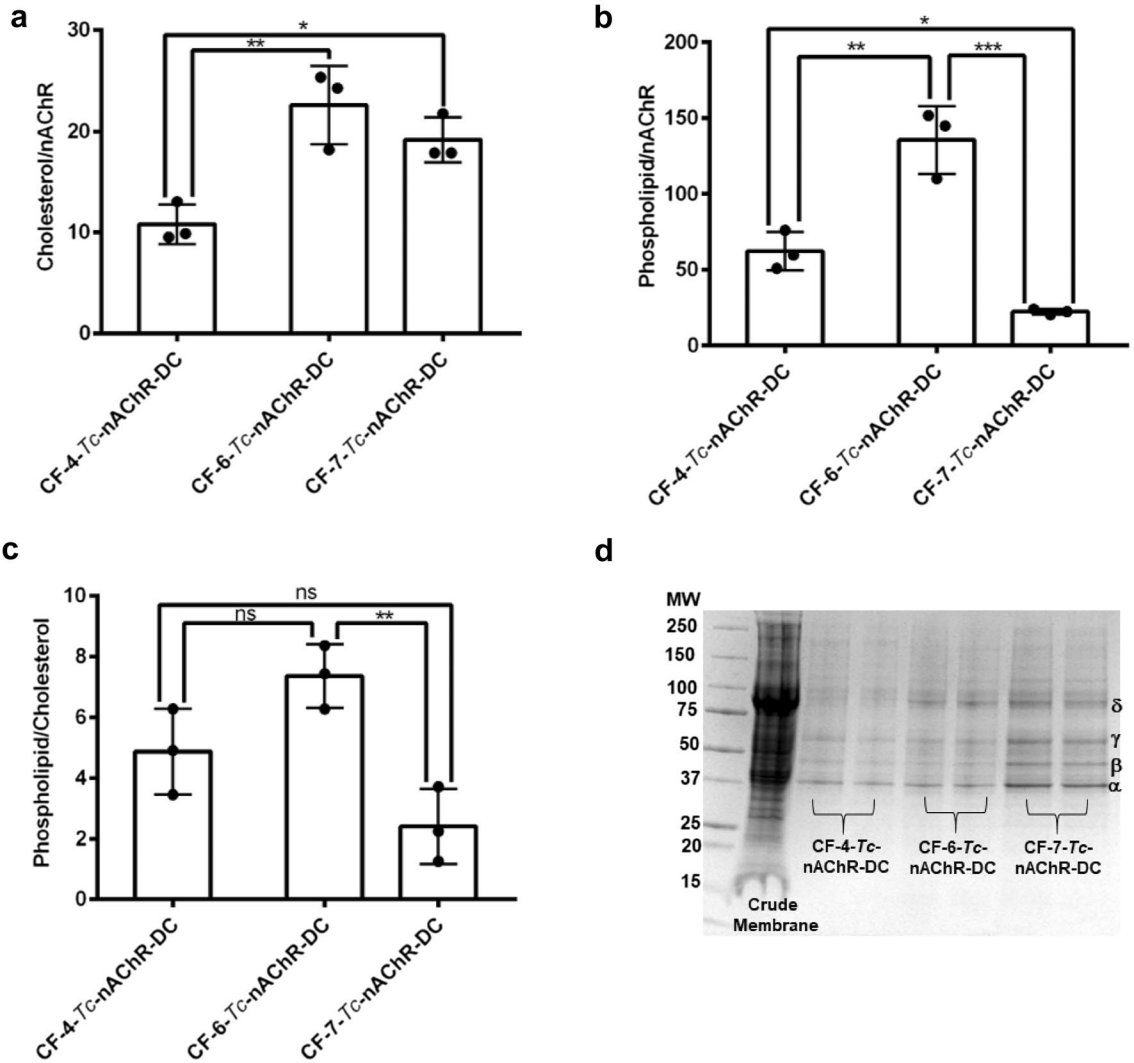
Effect of detergent in the number of molecules of phospholipids, cholesterol, and nAChR present in the CF-*Tc*-nAChR-DC**.** Affinity-purified CF-*Tc*-nAChR-DC for the three detergents were analyzed as mentioned in methods section for (**a**) cholesterol to nAChR ratio **(b**) phospholipid to nAChR ratio, (**c**) phospholipid to cholesterol ratio, and (**d**) SDS-PAGE for the crude membranes and the three CF-*Tc*-nAChR-DC. The data represent the mean ± SD of three (3) independent extractions for each experimental condition using one-way ANOVA followed by Tukey’s multiple comparison test, comparison to CF-4-*Tc*-nAChR-DC

Due to the possible delipidation by the detergent solubilization process of *Tc*-nAChR, the ratio of cholesterol/nAChR, phospholipid/nAChR, and phospholipid/cholesterol was determined for the three CF-*Tc*-nAChR-DCs studied here. Apparently, the CF detergents studied have a structure that helps to maintain cholesterol associated with the nAChR, and depends on the separation of the hexane ring from the head group in the CF family assayed (Baier et al. 2011; Maldonado-Hernández et al. 2020). The mean cholesterol/nAChR ratio measured was 10.8, 22.6, and 19.5 for CF-4, CF-6, and CF-7, respectively, Fig. 2a. However, the behavior of this family in its ability to maintain the phospholipid composition, which is critical for nAChR functionality, presents a variability with respect to the length of the ring-expanding aliphatic chain.

According to the results of the analysis of the ratio of phospholipids per nAChR molecule shown in Fig. 2b, extending the aliphatic chain by two carbons from CF-4 to CF-6 affect the phospholipids to nAChR ratio by more than two-fold. The CF-4 detergent produces a phospholipid/nAChR ratio quite similar to those previously reported for the native membrane nAChR, approximately 60 phospholipids per nAChR (Schmidpeter et al. 2022). However, an increase in the aliphatic chain by an odd number that positions the ring in a unique spatial conformation, as is the case of CF-6 versus CF-7, has a substantial effect on the delipidation of nAChR. The ratio of phospholipid/cholesterol for the three CF detergents studied here presents a similar range in ratio to the previous lipid-like detergent used in our lab, where CF-6 presents a ratio that is three times greater than that obtained for CF-7. However, CF-4 remains the best of the three detergents since it produces a ratio of approximately five phospholipid molecules per cholesterol molecule, which has been shown to satisfy the requirements for maintaining stability and functionality in *Tc*-nAChR-DC (Barrantes 2004; Hamouda et al. 2006a, b; Baier et al. 2011). The purity of both crude membranes and CF-*Tc*-nAChR-DC was accessed qualitatively by SDS-PAGE. All four nAChR subunits (α, β, γ, and δ) were resolved and migrated single bands. In addition to usual co-solubilized proteins rapsyn (43-kD), but also a much lower level of ATPase (100-kD) were observed, Fig. 2d. A careful inspection of the SDS gels indicates that all of the CF-nAChRs-DCs displayed the same degree of impurities, Fig. 2d.

### Effect of CF in the Functionality of the Tc-nAChR Using TEVC Technique

**Fig. 3:**
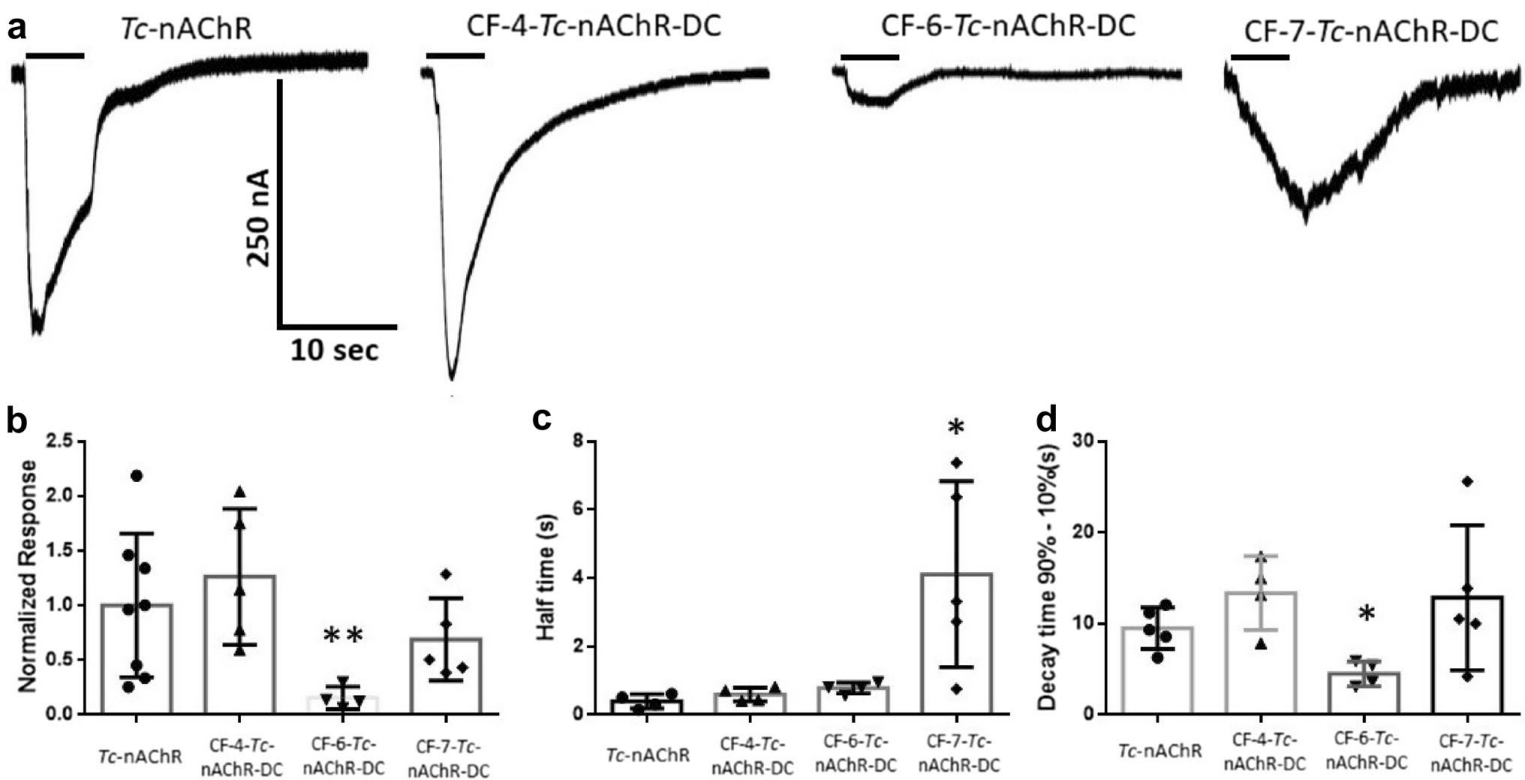
Macroscopic ion-channel functional assays for the crude membranes and solubilized and affinity-purified CF-*Tc*-nAChR-DCs. **A** Representative responses to Crude membrane; and *Tc*-nAChRs solubilized using CF-4; CF-6 and CF-7. Responses were evoked by a 5-s application of 100 µM ACh (represented by black bars). **B** CF-*Tc*-nAChR-DCs responses were normalized to the response of crude membranes. **C**–**D** The kinetics of the different CF-*Tc*-nAChR-DCs responses were compared to crude membrane preparations using the activation half-time (C) and the decay time (D); as measurements of activation and deactivation, respectively. The normalized responses, activation and deactivation kinetics of CF-*Tc*-nAChR-DCs were analyzed statistically using a t-test Mann Whitney comparing all the different CF-*Tc*-nAChR-DCs to crude membranes (Graph Pad Prism 6); *p < 0.05 and **p < 0.01

The TEVC experiments were done following our previously published protocol (Padilla-Morales et al. 2016). To evaluate the functional effects of the CF family of detergents, the CF-*Tc*-nAChR-DCs were injected into *Xenopus laevis* oocytes and compared to crude membrane extracts using a non-saturating concentration of acetylcholine (ACh) to activate the nAChR response and measured by TEVC. A 5-s application of 100 μM ACh in an oocyte injected with *Tc*-nAChR’s crude membrane resulted in a mean amplitude of − 247 nA response (− 247 ± 57 nA; *n* = 8), (see Fig. 3a). When the *Tc* crude membranes were solubilized with the CF detergents family followed by affinity-column purification and injected into oocytes, the mean amplitude responses evoked by ACh produced the following currents, for the CF-4*-Tc*-nAChR-DC (− 312 ± 69 nA, *n* = 5), CF-6*-Tc*-nAChR-DC (− 37 ± 13 nA, *n* = 4), and CF-7*-Tc*-nAChR (− 170 ± 42 nA, *n* = 5) (see Fig. 3a). The activation and deactivation kinetics of the different CF-*Tc*-nAChR-DCs were compared to crude membrane *Tc*-nAChRs using the activation half-time and the decay time (90%-10%). As shown in Fig. 3b the activation half-time was 0.4 ± 0.1 s in crude *Tc*-nAChRs membranes; 0.6 ± 0.1 s for CF-4-*Tc*-nAChR-DC; 0.8 ± 0.08 s for CF-64-*Tc*-nAChR-DC; and 4.1 ± 1.2 s for CF-74-*Tc*-nAChR-DC, (see Fig. 3c). In order to examine the deactivation kinetics, we analyzed the macroscopic current decay time of the crude and the *Tc*-nAChR-DC. Decay times were 9.5 ± 1.0 s for crude *Tc*-nAChRs membranes; 13.4 ± 2.0 s for CF-4-*Tc*-nAChR-DC; 4.5 ± 0.7 s for CF-6-*Tc*-nAChR-DC; and 12.85 ± 3.6 s for CF-7-*Tc*-nAChR, (see Fig. 3d).

## Discussion

Studies aimed at obtaining crystallographic structures of membrane proteins must overcome a series of obstacles, particularly, the challenge to obtain the protein of interest as pure as possible and in high yields. Moreover, the detergent selected for the solubilization process needs to produce a stable protein and help to keep the protein functionality. Choosing a suitable detergent that meets all the physical–chemical requirements to solubilize the membrane protein is one of the most challenging and critical tasks prior to the crystallization step. The detergent must intercalate into the membrane and extract the protein of interest and in turn produce a host environment for the protein. In the native membrane, the membrane protein is stabilized by the ring of lipids that interact directly with the hydrophobic belt of the protein and by the lateral pressure provided by the lipids and other proteins that make up the native bilayer. This lateral pressure is compromised in the detergent micelles and it depends on the physicochemical properties of the detergent. Therefore, the stability of the membrane protein in the detergent complex will depend on the level of preservation of the endogenous annular lipids, in other words, on achieving the least degree of delipidation.

For more than two decades, our laboratory has been given the task of finding the best conditions to produce stable and functional *Tc*-nAChR-DC for crystallization trials. Previous work in our laboratory studied the purity and stability of commonly used detergents for the production of solubilized and affinity-purified *Tc*-nAChRs-DC as a prelude to crystallization. In addition, we measured the functionality using the planar Lipid Bilayer Technique (Cherezov et al. 2008; Padilla-Morales et al. 2016). The experience gained over decades of work in our laboratory has shown that the characterization of nAChR ion-channel function using the TEVC technique in oocytes is more effective. In addition, the stability of the *Tc*-nAChR-αBTX Alexa 488-DC in LCP for phospholipid and cholesterol-analog detergents was assayed using FRAP (Padilla-Morales et al. 2015, 2016; Maldonado-Hernández et al. 2020). Our results showed that native lipid depletion occurred in all detergents within certain ranges, depending on the lipid-analogue detergent’s structure, triggering different degrees of stability and functionality. However, most of the lipid-like detergents maintain stability and support ion-channel function such as (3-[(3-Cholamidopropyl)-dimethylammonio]-1-propane sulfonate)] (CHAPS), n-Dodecylphosphocholine (FC12) n-tetradecylphosphocholine (FC-14), n-Hexadecylphosphocholine (LFC-16), and 3α,7α,12α-Trihydroxy-5β-cholan-24-oic acid (sodium cholate) (Asmar-Rovira et al. 2008; Padilla-Morales et al. 2015, 2016). In contrast, non-lipid-analog detergents such 6-Cyclohexyl-1-hexyl-β-d-maltoside (Cymal-6), n-Dodecyl-β-d-maltopyranoside (DDM), Lauryldimethylamine-N-oxide (LDAO) and n-Octyl-β-d-glucopyranoside (OG), Polyoxyethylene-(9)-dodecyl ether Anapoe-C12E9) and N,N′-bis-(3-d-Gluconamidopropyl cholamide) (BigCHAP) show decreased stability and significant reduction or loss of ion-channel function (Asmar-Rovira et al. 2008). Overall, these results indicate that the nAChR can be stable and functional in lipid-analog detergents or in detergents that retain moderate amounts of residual native lipids, while the opposite is true about non-lipid-analog detergents. These data highlight the importance of a careful biophysical characterization of the membrane protein-detergent complex (MP-DCs) for future structural studies (Hamouda et al. 2006a, b; Maldonado-Hernández et al. 2020; Delgado-Vélez et al. 2021).

In this study we took on the task of assessing the capacity of three short-chain lipid-like detergents containing a six-carbon ring at the end of the hydrophobic tail. All the detergents of the CF family assayed produced a considerable amount of protein, in the range of mg and reproducible under the same solubilization conditions. The CMC for CF-4, CF-6, and CF-7 are 8.45, 2.68, and 0.62 mM, respectively (see Table 1). Thus, CF-6 and CF-7 produce larger micelles and increase in aggregation number compared to CF-4 (Anandan and Vrielink 2016). As shown in Fig. 1, the CF-4 detergents produced the best *Tc*-nAChR-DC behavior in terms of its LCP fractional fluorescence recovery at the end of the study period, while its maximum recovery is in the range of some non-cyclofoscholine detergents studied in our laboratory, e.g., FC-12, FC-14 and FC-16, (Padilla-Morales et al. 2016). However, increasing the length of the aliphatic chain in these FC detergents from 12 to 16 carbons produced a substantial improvement in the value fractional fluorescence recovery. Still, this is not completely true for the CF detergent family studied. When increasing from four to six carbons in length (CF-4 to CF-6) the fractional fluorescence recovery at 30 days decreased by approximately 14% for CF-6 and 24% for CF-7. This behavior can be explained in view of the physicochemical properties of CF detergents and their capacity to interact with the cholesterol molecule. Based on examining the detergents and cholesterol structure, it is inevitable that the CF molecules are capable of establishing a stabilizing interaction by means of Van der Waals forces between the aliphatic chain of the detergent and the A, B, and C rings of the cholesterol molecule. Our results showed that both detergents CF-6 and CF-7 maintain approximately the same amount of cholesterol per nAChR in the *Tc*-nAChR-DC; however, CF-4 produced at least 50% less cholesterol per nAChR.

Although the number of molecules of cholesterol in the *Tc*-nAChR-DC for CF-6 and CF-7 does not significantly differ, CF-7 apparently produces some type of rejection against phospholipids as compared to CF-4 and CF-6 with a 2.8 and 6.1 decreased fold, respectively. This individual behavior translates into phospholipid/cholesterol ratios for CF-4 and CF-6 of approximately 5 and 7, which are similar to those reported for lipid-like detergent analog mentioned above (see Fig. 2). Studies carried out in the late 1980s where the effect of lipid composition on the functionality of the solubilized *Tc*-nAChR was determined suggested that at least a ratio of 45 lipids/nAChR should be present to observe activity (Marsh et al. 2002; Hamouda et al. 2006a). Furthermore, the amount of cholesterol present in model membranes that support nAChR functionality should be approximately 35 mol%, since this value is similar to that found in the native membranes *Tc*-nAChR-DC (Marsh et al. 2002). This implies that there must be at least three (3) sterol molecules per receptor subunit, although, this extrapolation is assuming that there is only one population of lipids due to the nAChR protein (annular lipid) and bulk lipids exchange rapidly. These previous studies did not present data about possible excess cholesterol and its effect in the receptor functionality. Previous studies in our laboratory determined the functionality and lipid composition of different *Tc*-nAChR-DCs formed by phospholipid-like detergents; however, none of the detergents increased the cholesterol/nAChR similar to the CF, especially the CF-6 and CF-7 which almost doubled the cholesterol/nAChR ratio. Apparently, the excess of cholesterol in CF-6-nAChR-DC and CF-7-nAChR-DC resulted in a decrease in the macroscopic response of the nAChR ion-channel. Specifically, the response value in CF-7 was reduced by one third, reaching 68% of the crude membrane response value, while the reduction in CF-6 was more significant, reaching an 85% reduction when compared to the crude membrane response (Fig. 3). These results are consistent with a previous study that demonstrated that a physiologically relevant increase in membrane cholesterol concentration produces a remarkable reduction in the macroscopic current responses of the *Tc*-nAChR as well as other neuronal nAChRs subtypes (Baez-Pagan et al. 2016. Likewise, the loss of phospholipids and the gain of cholesterol molecules in CF-7-Tc-nAChR-DC may be the explanation for its behavior with respect to its mobility in LCP and LCP: cholesterol mixture. We hypothesize that the spatial orientation and transmembrane location of the of the cyclohexane ring in CF-7 could result in a more efficient interaction with the cholesterol C ring in the LCP:cholesterol mixture over the other CF detergents studied. This could contribute to the lowest 5% increase in the diffusion coefficient of CF-7-Tc-nAChR-DC compared to CF-4-Tc-nAChR-DC and CF-6-Tc-nAChR-DC which increased their diffusion coefficient in LCP:cholesterol mixture by 10% and 33%, respectively.

While the Xenopus oocyte expression system is ideal for these types of studies, it is certainly not without drawbacks. Indeed, when doing these experiments, we found in most cases there is always a loss of functional activity except for the LFC-14 and LFC16 that were similar to the crude preparation (Padilla et al., 2016). In the preset study the nAChR-CF-4 complex gave a functional response that was similar to the crude. Is important to mention that the injection of nAChR-DCs containing 1.5 critical micellar concentration of a detergent, that results in a reduction on the viability of the oocytes. This reduction in viability is traduced in the observed loss of functional nAChR ion activity. We have previously noticed that some detergents showed a reduction on viability within the first 10 h making it hard for us to get TEVC responses (specially, cholesterol-analog detergents), but for the detergents used in the present study we recorded activity between 16–36 h. This time frame gave us a window in which we could maintain oocyte viability ensuring reliable responses. Also, it has been previously proposed that the reason for such loss in function could be due to the viscous nature of the membrane preparations, and that the fluid injected into the oocytes usually contain variable amounts of receptor-bearing membranes (Marsal et al., 1995). Furthermore, lack of these (acid-rich) phospholipids in the nAChR-DCs could also contribute to the reduced functional responses recorded in oocytes.

Interestingly, the CF-4*-Tc*-nAChR-DC that enclosed the largest lipid species (13) in total, displays the best functionality compared to CF-6*-Tc*-nAChR-DC and CF-7*-Tc*-nAChR-DC that enclosed only 6 and 9, respectively (see Table 2). Furthermore, functional assays of the solubilized *Tc*-nAChR-DC reconstituted in model membranes at different lipid to protein mole ratios showed a progressive decrease in receptor activity as the phospholipid/nAChR ratio decreased below 45. Also, this preparation produced an irreversible inactivation below a ratio of 20, this is the case of the CF-7*-Tc*-nAChR-DC (Jones and McNamee 1988; Quesada et al. 2016; Schmidpeter et al. 2022). Figure 2b presents the phospholipid to nAChR-DC ratio for the CF-4*-Tc*-nAChR-DC and CF-6*-Tc*-nAChR-DC and CF-7*-Tc*-nAChR-DC with ratio values of 62, 135, and 22, respectively. By correlating these values with the macroscopic current response produced by nAChR-DC injected into the membranes of *Xenopus* oocytes, we found that CF-4*-Tc*-nAChR-DC, which presented a phospholipids/nAChR ratio (62) in the range previously determined to be functional, was the only one of the three detergents studied that produced an adequate normalized macroscopic current response (Fig. 3). Compared to the CF-4*-Tc*-nAChR-DC, the CF-6*-Tc*-nAChR-DC and CF-7*-Tc*-nAChR-DC produced some response, but only 15% and 49%, respectively, relative to the crude membrane response value. Furthermore, the activation kinetics which were measured as activation half-time were significantly slower for the CF-7*-Tc*-nAChR-DC when compared to *Tc*-nAChR from crude membranes. Interestingly, neither CF-4*-Tc*-nAChR-DC nor CF-6*-Tc*-nAChR-DC nor had a significant effect on activation kinetics; however, when we looked at deactivation kinetics using the decay time (90%–10%) we found that it was significantly faster for CF-6*-Tc*-nAChR-DC. Consistent with the idea that CF-4*-Tc*-nAChR-DC is able to maintain the normal function of *Tc*-nAChR all the parameters measured using TEVC were not significantly different than values obtained from crude membrane preparations. Also, an increase in the phospholipid/nAChR ratio reduced the functional response of the nAChR, as shown in the case of CF-6*-Tc*-nAChR-DC which displayed a ratio of 135.

Overall, the present study demonstrates that the selection of a detergent to solubilize a membrane protein is an empiric experiment, and one of the critical factors is the composition of lipids that remain after extraction in the MP-DCs. The structure and physicochemical properties of the detergent sculpts the composition of the lipids that remains in the MP-DC by selectively including and excluding certain critical lipids species. Most important, the lipid composition that remains in the MP-DC affects the purity, functionality, and stability of the MP. The present study reveals that for the *Tc* (muscle-type) nAChR-DC certain lipid species such as (SM (d16:1/18:0); PC (18:2/14:1); PC (14:0/18:1); PC ((16:0/18:1); PC (20:5/20:4) and PC (20:4/20:5)) are crucial to retain ion-channel functionality. The interpretation of these results can be perceived as a discreet lipid composition that support ion-channel function; however, from a broader and more complex neurophysiological perspective, we can hypothesize that each nAChR neuronal subtype might have specific lipid species requirements to maintain their diverse ion-channel properties and ultimately the cholinergic neurotransmission in the central nervous system.
